# Inhibition of PD-1 Alters the SHP1/2-PI3K/Akt Axis to Decrease M1 Polarization of Alveolar Macrophages in Lung Ischemia–Reperfusion Injury

**DOI:** 10.1007/s10753-022-01762-6

**Published:** 2022-11-11

**Authors:** Xiaojing He, Jingyuan Xiao, Zhao Li, Mengling Ye, Jinyuan Lin, Zhen Liu, Yubing Liang, Huijun Dai, Ren Jing, Fei Lin

**Affiliations:** 1 Department of Anesthesiology, Guangxi Medical University Cancer Hospital, Nanning, China; 2 Guangxi Clinical Research Center for Anesthesiology, Nanning, China; 3 Guangxi Engineering Research Center for Tissue & Organ Injury and Repair Medicine, Nanning, China; 4 Guangxi Key Laboratory for Basic Science and Prevention of Perioperative Organ Dysfunction, Nanning, China; 5 Department of Experimental Research, Guangxi Medical University Cancer Hospital, Nanning, China

**Keywords:** lung ischemia–reperfusion injury, PD-1, macrophage polarization, inflammation, PI3K/Akt pathway.

## Abstract

Polarization of alveolar macrophages (AMs) into the M1 phenotype contributes to inflammatory responses and tissue damage that occur during lung ischemia–reperfusion injury (LIRI). Programmed cell death factor-1 (PD-1) regulates polarization of macrophages, but its role in LIRI is unknown. We examined the role of PD-1 in AM polarization in models of LIRI *in vivo* and *in vitro*. Adult Sprague–Dawley rats were subjected to ischemia–reperfusion with or without pretreatment with a PD-1 inhibitor, SHP1/2 inhibitor, or Akt activator. Lung tissue damage and infiltration by M1-type AMs were assessed. As an *in vitro* complement to the animal studies, rat alveolar macrophages in culture were subjected to oxygen/glucose deprivation and reoxygenation. Levels of SHP1/2 and Akt proteins were evaluated using Western blots, while levels of pro-inflammatory cytokines were measured using enzyme-linked immunosorbent assays. Injury upregulated PD-1 both *in vivo* and *in vitro*. Inhibiting PD-1 reduced the number of M1-type AMs, expression of SHP1 and SHP2, and levels of inflammatory cytokines. At the same time, it partially restored Akt activation. Similar results were observed after inhibition of SHP1/2 or activation of the PI3K/Akt pathway. PD-1 promotes polarization of AMs to the M1 phenotype and inflammatory responses through the SHP1/2-PI3K/Akt axis. Inhibiting PD-1 may be an effective therapeutic strategy to limit LIRI.

**Supplementary Information:**

The online version contains supplementary material available at 10.1007/s10753-022-01762-6.

## INTRODUCTION

Lung ischemia–reperfusion injury (LIRI) is a complex pathophysiological process that often occurs in patients with lung disease or following surgery. For example, severe LIRI can occur after lung transplantation, leading to systemic hypoxemia and multiple organ failure, which are major causes of postoperative lung failure and death [1, 2]. Thus, finding appropriate therapeutic targets against LIRI is imperative for improving patient outcomes.

Alveolar macrophages (AMs) play a critical role in acute pulmonary inflammation [3]. In response to various stimuli, AMs express different phenotypes, such as pro-inflammatory M1 and anti-inflammatory M2 phenotypes [4]. Activated M1 macrophages express CD11c, CD16, and CD86, as well as pro-inflammatory cytokines such as TNF-α, IL-1β, and IL-6. The M2-type macrophage expresses elevated levels of Arginase-1 (Arg-1) and CD206, as well as anti-inflammatory cytokines such as IL-4 and IL-1 [5–7]. M1 macrophages protect from infection and also help induce inflammation. M2-type macrophages, on the other hand, inhibit inflammation and promote tissue repair. Regulating macrophage phenotypes is crucial to maintaining immune function and homeostasis [8].

Programmed cell death factor-1 (PD-1) is a member of the immunoglobulin superfamily expressed specifically on activated T cells, B cells, natural killer cells, dendritic cells, and macrophages [9, 10]. The cytoplasmic tail of PD-1 contains an immunoreceptor tyrosine-based inhibitory motif (ITIM) and an immunoreceptor tyrosine-switch motif (ITSM) [11]. The ITSM domain of PD-1 can recruit the Src homology region two domain-containing phosphatases (SHPs) 1 and 2 in order to exert immunosuppressive effects, inhibition of T cell proliferation, and activation [9, 12]. Ischemia–reperfusion in isolated hearts significantly upregulates PD-1 in cardiomyocytes, aggravating their injury [13]. Furthermore, PD-1 plays an important role in immune regulation and is involved in the progression of disease by regulating macrophage polarization [14, 15].

The PI3K/Akt signaling pathway is involved in regulating macrophage polarization by TSC1/2 mTOR [16]. Studies have shown that PI3K/Akt signaling pathway protects organs from ischemia–reperfusion (I/R) injury [17, 18]. Additionally, PD-1 recruits SHP2 to its cytoplasmic tail, leading to inactivation of the PI3K/Akt signaling pathway and promotion of macrophage apoptosis [19]. However, it is not clear yet whether PD-1 affect PI3K/Akt during lung I/R.

Therefore, we hypothesized that PD-1 might contribute to LIRI by inducing the polarization of AMs to the M1-type, and that inhibition of SHP1/2 might activate PI3K/Akt, thereby reduce M1-type polarization in favor of the M2-type and reducing lung damage after I/R. In this study, *in vivo* and *in vitro* models were used to investigate the role of PD-1 in LIRI and the underlying mechanisms.

## MATERIALS AND METHODS

### Reagents and Antibodies

BMS202 (PD-1 inhibitor), SC79 (Akt activator), and NSC87877 (SHP1/2 inhibitor) were purchased from Selleck Chemicals (Houston, TX, USA). Enzyme-linked immunosorbent assay (ELISA) kits for TNF-α, IL-6, and IL-1β were purchased from Elabscience Biotechnology (Wuhan, China). Antibodies against HIF-1α, SHP1, SHP2, phosphorylated Akt (p-Akt), Akt, and β-actin were purchased from Cell Signaling Technology (Danvers, MA, USA). Antibodies against PD-1, CD11c, CD16, CD86, and CD86/PE were from Bioss (Beijing, China). Fluorophore-labeled goat anti-rabbit secondary antibody (Alexa Fluor 594), goat anti-rabbit immunoglobulin horseradish peroxidase (IgG-HRP), bovine serum albumin (BSA), and the bicinchoninic acid (BCA)-based protein assay were purchased from Beyotime (Shanghai, China). The 2-step plus Poly-HRP Anti Mouse/Rabbit IgG Detection System kit was from Solarbio Life Sciences (Beijing, China). TRIzol reagent was purchased from Invitrogen (Carlsbad, CA, USA); PrimeScript™ RT Kit, from Takara (Osaka, Japan); fetal bovine serum (FBS); and Ham’s F-12 K (Kaighn’s) medium, from Gibco (Carlsbad, CA, USA).

### Animals

Adult male Sprague–Dawley rats (220–250 g, 6–8 weeks) were purchased from the Animal Center of Guangxi Medical University (Nanning, China). Rats were maintained in a specific pathogen-free facility at 40–70% ambient humidity, temperature of 18–26 °C, and 12-h day-night cycles. Animals had ad libitum access to sterilized drinking water and food. All animal protocols complied with the Animal Guide of the Institutional Animal Care and Use Committee of Guangxi Medical University (Nanning, China).

### LIRI Model

The rat LIRI model was established as previously reported [20]. The roles of PD-1, SHP1/2, and Akt were studied in three separate cohorts of rats. All rats were randomly divided into groups (*n* = 3 per group). The first cohort was divided into four groups: Control, BMS202, I/R, and I/R + BMS202. The second cohort was divided into the following four groups: Control, SC79, I/R, and I/R + SC79. The third cohort of rats was divided into four groups: Control, NSC87877, I/R, and I/R + NSC87877. Rats in the control groups did not receive drugs and surgery, and were sacrificed by bloodletting through the common carotid artery. Rats in each I/R group underwent thoracotomy, and the left hilus pulmonis was clamped for 1 h with vascular forceps. The clamps were removed and the rats were sacrificed by carotid exsanguination after 2 h, 6 h, 12 h, 24 h, and 72 h after allowing reperfusion.

Animal serum and left lung tissues were collected and stored in liquid nitrogen for subsequent testing. Rats in the BMS202 group were given 5 mg/kg BMS202 by oral gavage [21] for 3 h and then sacrificed. Rats in the I/R + BMS202 group were given 5 mg/kg BMS202 by oral gavage at 1 h before mock surgery, and the rest of the operations were the same as the IR-2 h group. Rats in the SC79 group were given 40 mg/kg SC79 by intraperitoneal injection [22] for 3 h and then sacrificed; Rats in the I/R + SC79 group were given 40 mg/kg SC79 by intraperitoneal injection at 1 h before mock surgery, and the rest of the operations were the same as the IR-2 h group. Rats in the NSC87877 group were given 10 mg/kg NSC87877 by oral gavage [23] for 3 h and then sacrificed. Rats in the I/R + NSC87877 group were given 10 mg/kg NSC87877 by oral gavage at 1 h before mock surgery, and the rest of the operations were the same as the IR-2 h group.

### Hematoxylin–Eosin Staining

Lung tissue specimens were fixed in 4% paraformaldehyde, embedded in paraffin, and cut into slices 5–8 μm thick. Samples were stained with hematoxylin and eosin (H&E), and observed under a light microscope (CX23, Olympus, Japan). The degree of lung tissue injury was scored as described [24] based on the following: aggregation or infiltration of inflammatory cells in vessel walls or air spaces (1 = only wall, 2 = few cells in air space, 3 = intermediate, or 4 = severe [air space congested]), hyaline membrane formation and interstitial congestion in the lung (1 = normal lung, 2 = moderate [> 25% of the lung section], 3 = intermediate [25–50% of the lung section], or 4 = severe [> 50% of the lung section]), and presence (1) or absence (0) of hemorrhage. The score for each criterion was summed to obtain the total score for each sample.

### Transmission Electron Microscopy

Lung tissue samples were sliced into pieces approximately 1 mm^3^ and fixed in 3% glutaraldehyde for more than 2 h, followed by 1% osmic acid for 1–2 h, and dehydrated in different concentrations of acetone before being embedded in resin. Samples were cut into ultrathin sections using an ultramicrotome and analyzed on a transmission electron microscope (Hitachi H-7560, Tokyo, Japan).

### Lung Wet-to-Dry Ratio

The lung wet-to-dry ratio was measured to assess the extent of pulmonary edema. Immediately after collecting the sample, the wet weight of the left lung tissue was weighed. The tissue was incubated at 60 °C for at least 96 h and weighed again. The wet-to-dry ratio was calculated [20].

### Immunohistochemical Staining

Lung tissue was embedded in paraffin and sliced into 5–8 μm thick sections. Samples were then dewaxed with xylene, dehydrated through graded ethanol solutions, incubated in 3% hydrogen peroxide to inactivate endogenous peroxidases, and subjected to antigen retrieval at pH 6.0 under high pressure. The sections were blocked with 3% hydrogen peroxide for 10 min, then incubated overnight at 4 °C with primary antibodies against CD11c (1:500), CD16 (1:500), and CD86 (1:500). The sections were exposed to biotin-labeled goat anti-rabbit IgG secondary antibody from the SP (mouse/rabbit IgG)-POD kit according to the manufacturer’s instructions.

Expression of macrophage surface markers was visualized using a light microscope (CX23, Olympus, Japan). Staining was quantified using Image-Pro-Plus (Media Cybernetics, Silver Spring, MD, USA). Five randomly selected fields of view were used to calculate the average immunoreactive score as follows [25]: staining intensity × percentage of positive cells. Staining intensity was categorized as 0 for negative; 1, weak; 2, moderate; or 3, strong. Percentage of positive cells was categorized as 0 for negative; 1, 10% positive cells; 2, 11–50% positive cells; 3, 51–80% positive cells; or 4, more than 80% positive cells.

### Flow Cytometry

AMs from the left lung tissue were extracted and diluted into a single-cell suspension. Cells were stained with PE-conjugated anti-CD86 antibodies at 4 °C in the dark for 30 min, followed by washing with phosphate-buffered saline (PBS). Expression of CD86 was detected by FACSCalibur™ flow cytometry (BD Biosciences, San Jose, CA, USA), and data were analyzed using Flow Jo software (Tree Star, San Carlos, CA, USA).

### *In Vitro *Oxygen/Glucose Deprivation and Reoxygenation (OGD/R) Model

The rat alveolar macrophage cell line NR8383 was purchased from ScienCell Research Laboratories (San Diego, CA, USA). The cells were routinely cultured in Ham’s F-12 K (Kaighn’s) medium containing 15% FBS at 37 °C in 95% air and 5% CO_2_. Prior to OGD/R, NR8383 cells were pre-treated with 1 µM BMS202 for 30 min [26, 30], 10 µM SC79 for 60 min [27], or 10 µM NSC87877 for 30 min [28].

Then NR8383 cells were subjected to OGD/R as described [29] in order to serve as an *in vitro* model of ischemia–reperfusion. Briefly, NR8383 cells were washed three times with glucose-free PBS pre-warmed to 37 °C, then fed serum-free and glucose-free Ham’s F-12 K medium, and finally cultured for 1 h at 37 °C in a Whitley H35 Hypoxystation (Don Whitley Scientific, Bingley, UK) in an atmosphere of 1% O_2_, 5% CO_2_, and 94% N_2_. Cells were allowed to recover for 2 h at 37 °C in glucose-containing Ham’s F-12 K medium in an atmosphere of 5% CO_2_ and 95% O_2_. Normoxic control cells were incubated at 37 °C in a humidified atmosphere composed of 5% CO_2_ and 95% O_2_.

### ELISAs

Homogenized fresh lung tissue or NR8383 cells were lysed with RIPA and centrifuged at 12,000 rpm for 15 min at 4 °C. The supernatant was assayed for TNF-α, IL-1β, and IL-6 using ELISA according to the manufacturer’s instructions.

### Western Blot Assay

Fresh lung homogenate from a single rat or washed NR8383 cells were lysed in RIPA buffer containing protease inhibitor cocktail and phosphatase inhibitor cocktail (Beyotime, Shanghai, China). After quantifying the protein concentration with a BCA kit, equal amounts of protein were fractionated on a 10–15% sodium dodecyl sulfate–polyacrylamide gel, then transferred to a 0.22-μm polyvinylidene fluoride membrane at 4 °C. After blocking with 5% BSA for 1 h, the membrane was incubated on a shaker at 4 °C overnight with primary antibodies against PD-1 (1:500), HIF-1α (1:1000), SHP1 (1:1000), SHP2 (1:1000), p-Akt (1:2000), Akt (1:2000), and β-actin (1:1000). The membrane was washed, then incubated with HRP-conjugated anti-rabbit secondary anti-IgG for 1 h at room temperature. Bands were visualized using enhanced chemiluminescence (Beyotime, Shanghai, China) reagent, then imaged using a Bio-Rad system (Hercules, CA, USA). Levels of target proteins under different treatment conditions were normalized to those from the corresponding Control group.

### Real-Time Quantitative PCR (RT-qPCR)

Total RNA was extracted from lung tissues and NR8383 cells using TRIzol, and total RNA (1 μg) was used in a 20 μL reverse transcription reaction with the PrimeScript™ RT Kit according to the manufacturer’s instructions. The cDNA was amplified using the following primers: PD-1 forward, 5′-CCGCTTCCAGATCGTACAACT-3′; PD-1 reverse, 5′-AGACTCCTATCTGCCTCACT-3′; GAPDH forward, 5′-CTATCGGCAATGAGCGGTTCC-3′; and GAPDH reverse, 5′-TGTGTTGGCATAGAGGTCTTTACG-3′. Gene expression was quantified using the 2^−ΔΔCt^ method and normalized to the expression of GAPDH.

### Immunofluorescence Staining

NR8383 cells were fixed with 4% paraformaldehyde at 4 °C for 1 h, washed with PBS three times, permeabilized with 0.2% Triton X-100 for 25 min, and then blocked with 5% BSA for 20 min. The samples were incubated with anti-CD86 antibody (1:500) at 4 °C for at least 12 h, then washed with PBS three times for 3 min each time. The cells were stained with Alexa Fluor 594-conjugated goat anti-rabbit secondary antibody, incubated at 25 °C in the dark for 50 min, and then washed with PBS three times for 3 min each time. Finally, nuclei were stained with 4-diimide-2-phenylindole (DAPI; Solarbio Life Sciences). Staining was observed under a fluorescence microscope (Olympus BX51, Tokyo, Japan).

### Statistical Analysis

Data were expressed as mean ± SD and analyzed using SPSS 22.0 (IBM, Armonk, NY, USA). Data were plotted using GraphPad Prism 5.0 (GraphPad Software, San Diego, CA, USA). Comparisons among multiple groups were analyzed by one-way analysis of variance, followed by the Tukey test for intergroup comparisons. Differences associated with *P* < 0.05 were considered statistically significant.

## RESULTS

### Expression of PD-1 Increases After Lung I/R

**Fig. 1:**
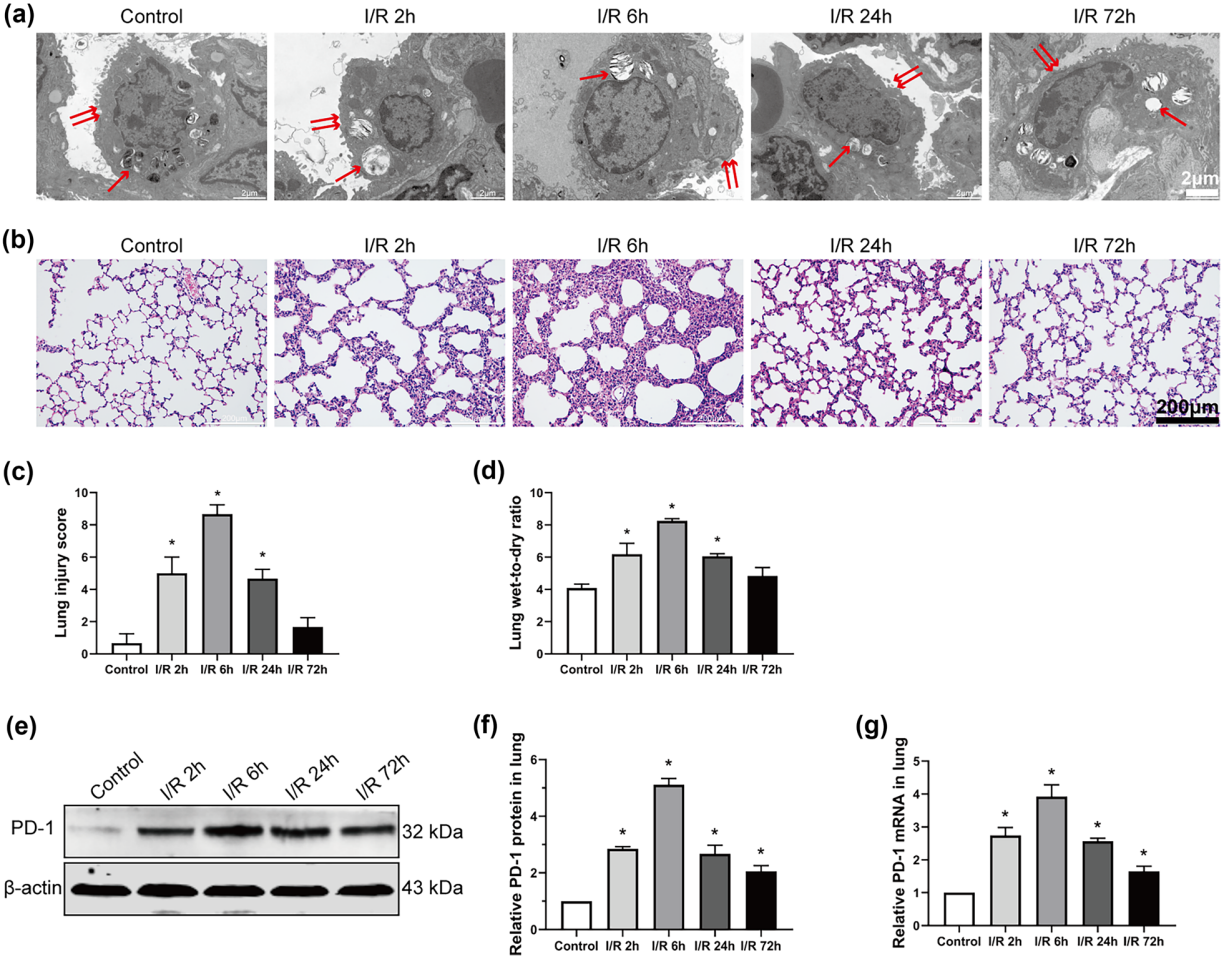
Expression of PD-1 increases after lung I/R. **a** Ultrastructural changes were assessed using TEM. The single arrow represents the lamellar body, and the double arrows represent the microvilli. Scale bar, 2 μm. **b** Morphological changes in the control and the I/R groups, as observed after the H&E stain. Scale bar, 200 μm. **c** Lung injury score. **d** Wet/dry ratios in lung tissue. **e** and **f** Western blot images and abundance of PD-1 in lung tissue. **g** Levels of PD-1 mRNA in lung tissue (*n* = 3 per group, mean ± SD, *P < 0.05 vs control group).

We established a rat model of LIRI and observed the ultrastructural changes in the lung tissue. As shown in Fig. 1(a), the ultrastructure of lung tissue in the control group was as expected, with more and clear microvilli and lamellar bodies. The I/R groups, however, showed severe structural changes, including decreased microvilli and lamellar bodies in type II epithelial cells. Symptoms of damage first appeared at 2 h after I/R and became more severe by 6 h, including almost complete loss of type II epithelial microvilli and lamellar corpuscles. The I/R 72-h group showed an ultrastructural recovery of lung tissue.

A morphological analysis of lung tissue was performed using H&E staining. The most serious damage was observed in the I/R 6-h group, which included pulmonary edema, alveolar collapse, increasing septal thickness, and extensive infiltration of inflammatory cells (Fig. 1b). In contrast, attenuated lung tissue injury was found in the I/R 72-h group. The pathological score of lung injury (Fig. 1c) was initially higher in the I/R 2-h group than in the control group, and the highest score was found in the I/R 6-h group. Finally, the wet-to-dry ratio of lung tissue was used to evaluate the degree of pulmonary edema and inflammatory exudation (Fig. 1d). In the I/R 6-h group, the wet-to-dry ratio was the highest. The above results indicated that we successfully established a rat LIRI model.

We then evaluated the expression of PD-1 at 2, 6, 24, and 72 h after I/R. Western blotting (Fig. 1e, f) and RT-qPCR (Fig. 1g) revealed that the expression of PD-1 was significantly upregulated in lung tissue across all timepoints.

### Inhibition of PD-1 Attenuates Lung Injury and Inflammation *In Vivo*

**Fig. 2:**
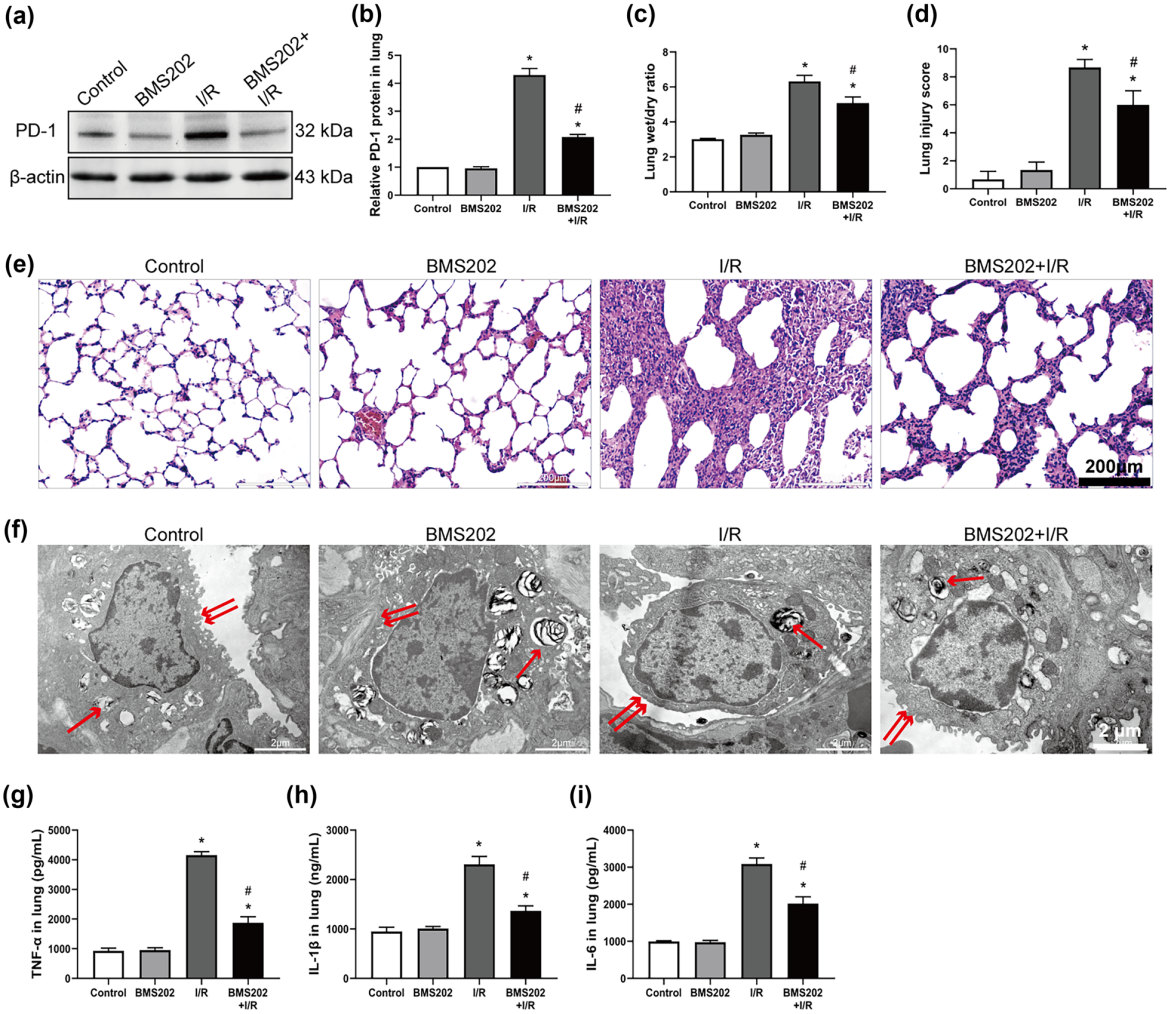
Inhibition of PD-1 attenuates lung injury and inflammation after lung I/R *in vivo*. **a** and **b** Western blot images and abundance of PD-1 in lung tissue. **c** Wet/dry ratio in lung tissue. **d** Lung injury score. **e** H&E staining images of lung tissue. Scale bar, 200 μm. **f** Ultrastructural changes observed by TEM: lamellar body (single arrow) and microvilli (double arrow). Scale bar, 2 μm. **g–i** Levels of TNF-α, IL-1β, and IL-6 in lung tissue (*n* = 3 per group, mean ± SD, *P < 0.05 vs control group, ^#^*P* < 0.05 vs I/R group).

Since PD-1 was already significantly upregulated by 2 h in the *in vivo* LIRI model, we chose this timepoint for the remaining experiments. We examined the role of PD-1 in LIRI by inhibiting it with BMS202. Pretreatment with BMS202 significantly reduced PD-1 expression (Fig. 2a, b) and the wet-to-dry ratio (Fig. 2c), and it protected against I/R-induced lung injury, as reflected in a significantly lower pathological lung injury score and less severe alterations of pulmonary morphology (Fig. 2d, e). In LIRI rats pretreated with BMS202, ultrastructural damage to lung tissue was significantly reduced in comparison to the I/R group, including a greater preservation of lamellar corpuscles and cytoplasm microvilli at the cell membrane surface (Fig. 2f). BMS202 treatment also dramatically reduced levels of inflammatory factors (Fig. 2g–i).

### Inhibition of PD-1 Reduces M1-Type AM Polarization *In Vivo*

**Fig. 3:**
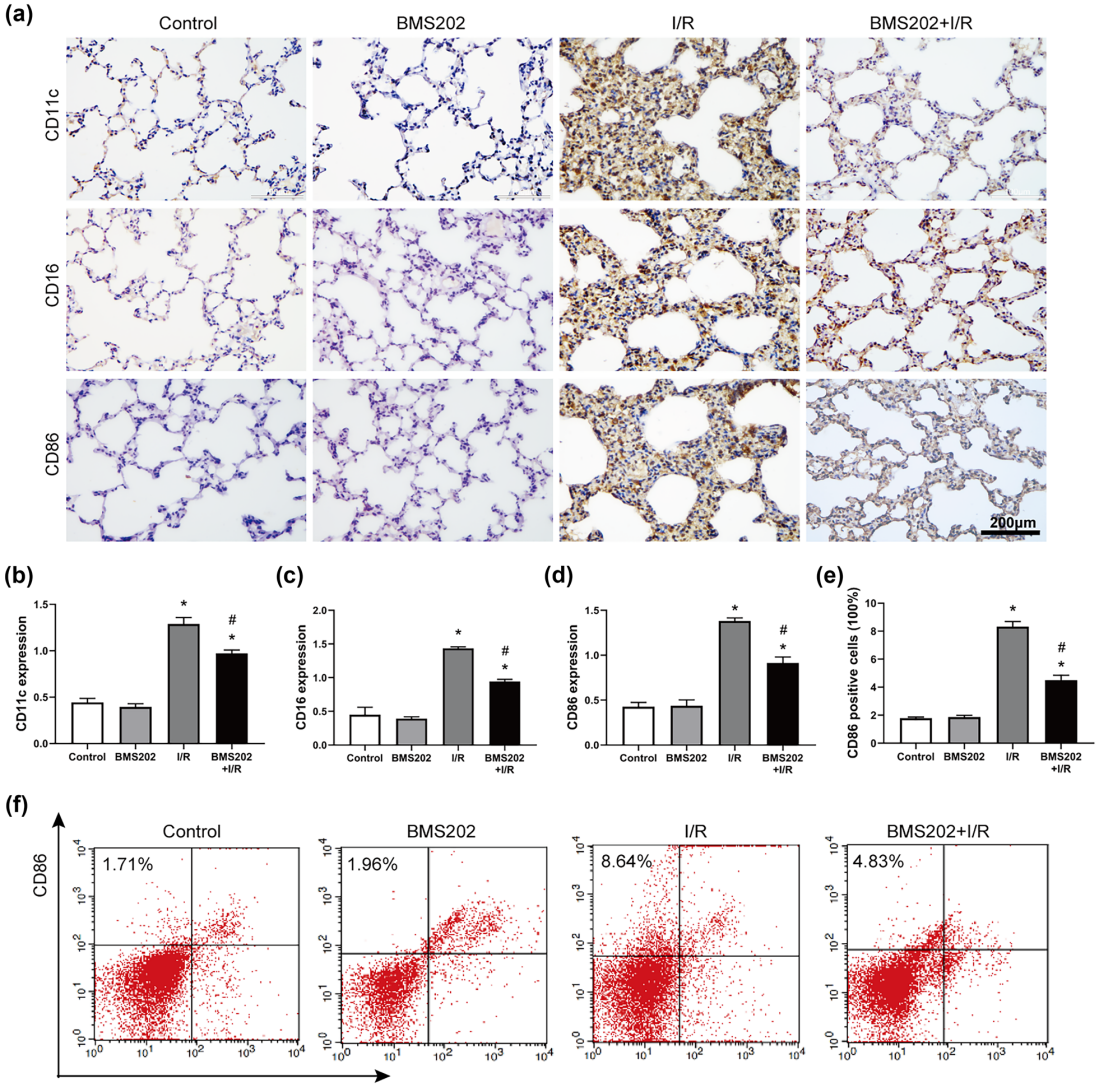
Inhibition of PD-1 reduces M1-type AM polarization induced by lung I/R *in vivo*. **a** Immunohistochemistry of CD11c, CD16, and CD86 in lung tissue. Scale bar, 200 μm. **b–d** Quantification of CD11c, CD16, and CD86 immunohistochemical staining. **e** Quantitative analysis of CD86-positive AMs in lung tissue. **f** CD86 expression in lung tissue was detected by flow cytometry (*n* = 3 per group, mean ± SD, *P < 0.05 vs control group, ^#^*P* < 0.05 vs I/R group).

Flow cytometry and immunohistochemical staining were used to investigate the effect of PD-1 on macrophage polarization in lung tissue. Pretreatment with BMS202 partially reversed I/R-induced upregulation of CD11c, CD16, and CD86, all markers of M1-type AMs (Fig. 3a–d). Consistently, CD86-positive AMs were significantly less abundant in the I/R + BMS202 group than in the I/R group, based on immunohistochemistry (Fig. 3e, f).

### Inhibition of PD-1 Reduces M1-Type AM Polarization and Inflammation *In Vitro*

**Fig. 4:**
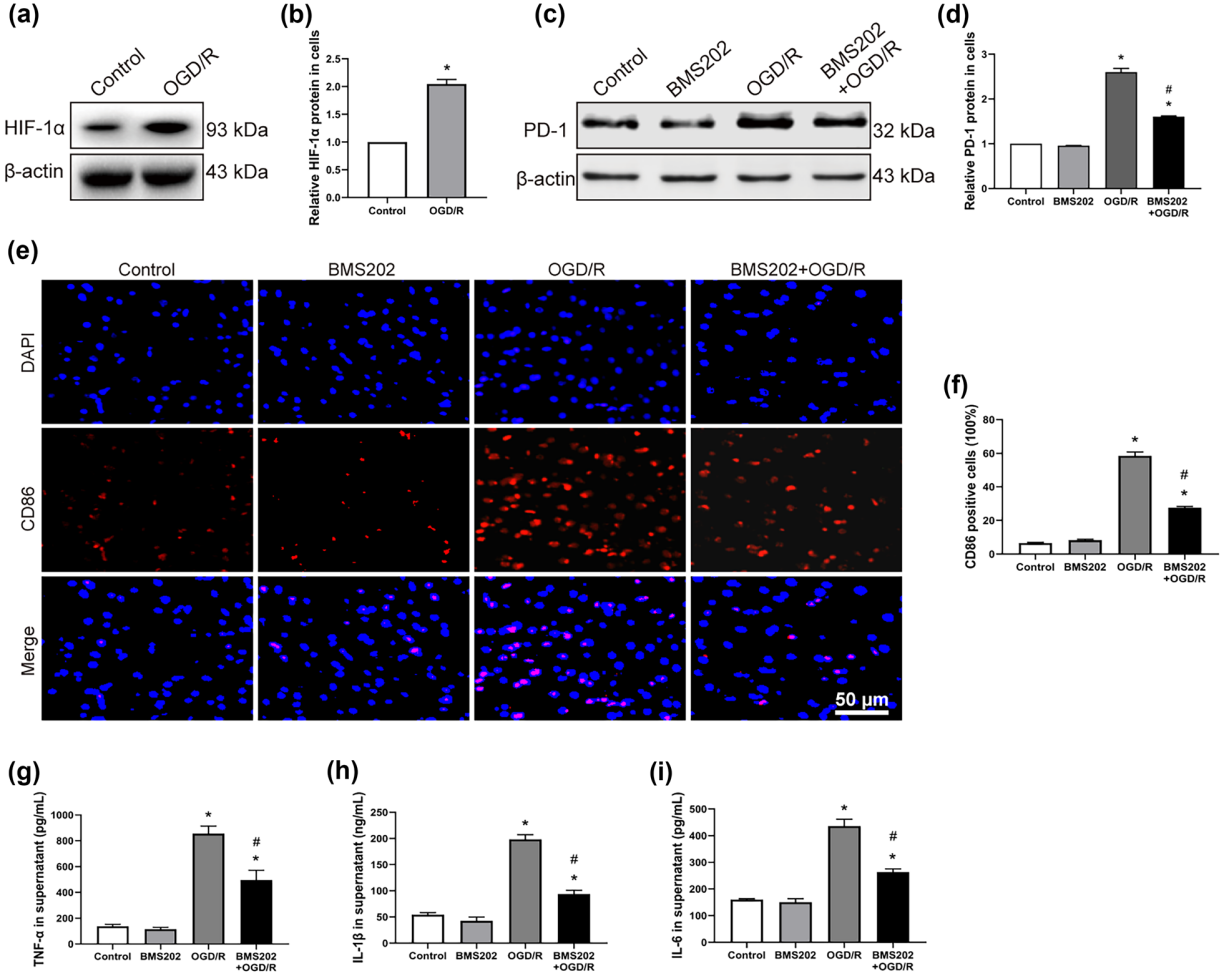
Inhibition of PD-1 reduces M1-type AM polarization and inflammation induced by OGD/R *in vitro*. **a** and **b** Western blot images and abundance of HIF-1α in NR8383 cells. **c** and **d** Western blot images and abundance of PD-1 in NR8383 cells. **e** Immunofluorescence staining of NR8383 cells. CD86 on the cell membrane was stained in red, and the nucleus was stained in blue. Scale bar, 50 μm. **f** Quantification of CD86-positive AM cells by immunofluorescence. **g–i** Levels of TNF-α, IL-1β, and IL-6 in cell supernatant (*n* = 3 per group, mean ± SD, *P < 0.05 vs control group, ^#^*P* < 0.05 vs OGD/R group).

We further investigated the effect of PD-1 on the polarization of NR8383 AMs in the OGD/R model. First, we checked the expression of HIF-1α to verify that we successfully induced hypoxia in the cells [20]. OGD/R dramatically increased HIF-1α expression (Fig. 4a, b), confirming that our model was successful. OGD/R increased PD-1 expression, and BMS202 suppressed this increase (Fig. 4c, d), consistent with the findings *in vivo*. OGD/R also significantly increased the number of CD86-positive AMs, and BMS202 suppressed this increase (Fig. 4e, f). Similarly, OGD/R markedly elevated levels of TNF-α, IL-1β, and IL-6, while pretreatment with BMS202 led to much smaller increases (Fig. 4g–i).

### Inhibition of PD-1 Reduces SHP1 and SHP2 Expression and Promotes High Activity of the PI3K/Akt Pathway *In Vivo* and *In Vitro*

**Fig. 5:**
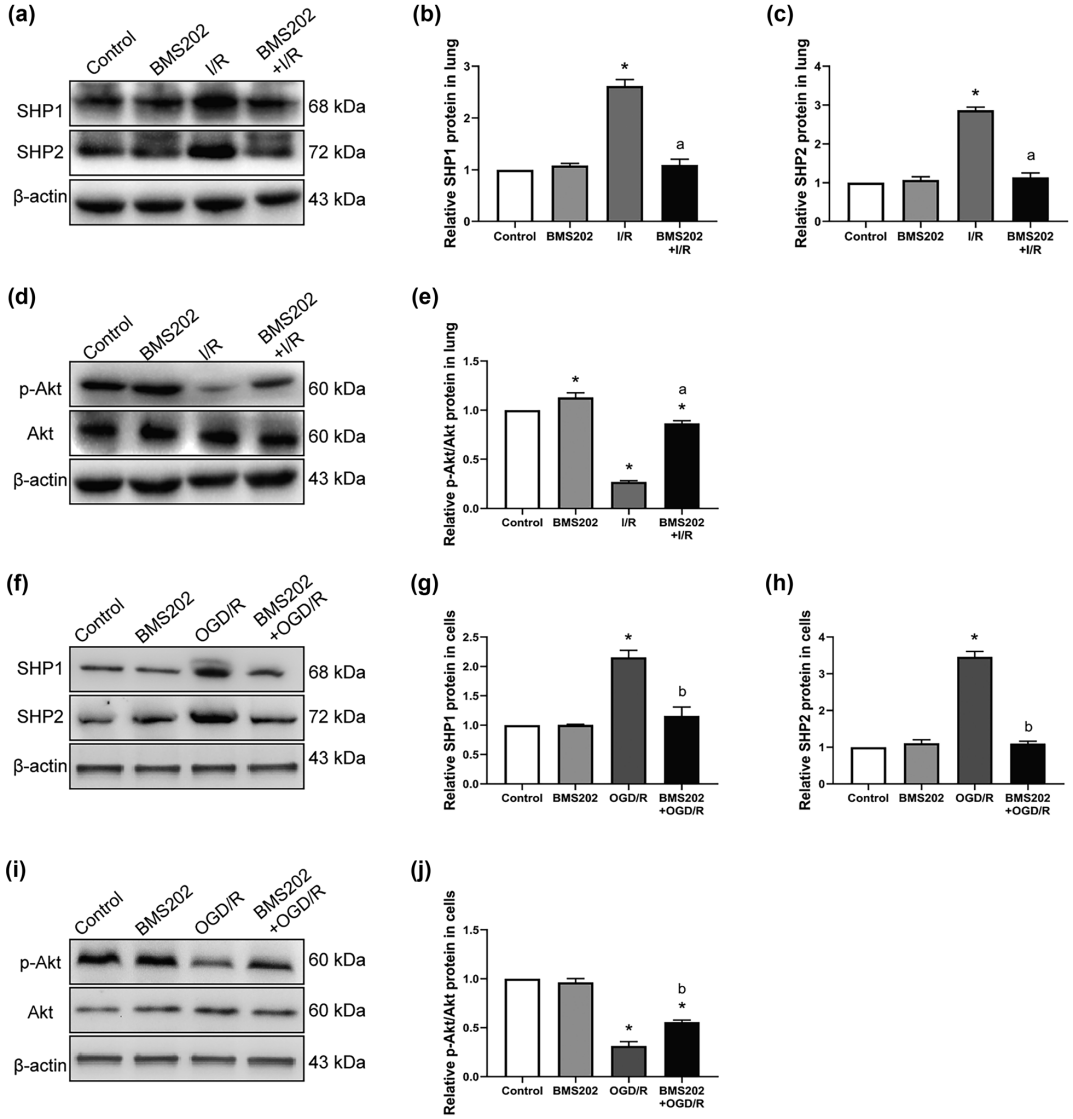
Inhibition of PD-1 reduces SHP1 and SHP2 expressions and restores the low activity of the PI3K/Akt pathway both *in vivo* and *in vitro*. **a–c** Western blot images and abundance of SHP1 and SHP2 in lung tissue. **d** Western blot images of p-Akt and Akt in lung tissue. **e** Relative abundance of p-Akt and Akt in lung tissue. **f–h** Western blot images and abundance of SHP1 and SHP2 in NR8383 cells. **i** Western blot images of p-Akt and Akt in NR8383 cells. **j** Relative abundance of p-Akt and Akt in NR8383 cells (*n* = 3 per group, mean ± SD, *P < 0.05 vs control group, ^a^*P* < 0.05 vs I/R group, ^b^*P* < 0.05 vs OGD/R group).

PD-1 recruits SHP1 and SHP2 to induce immunosuppression. I/R dramatically upregulated expressions of SHP1 and SHP2 in rat lung, which BMS202 partially reversed (Fig. 5a–c). Given that PD-1 and activation of the PI3K/Akt pathway seem to have conflicting roles in AM polarization, we measured activated Akt (p-Akt) and total Akt after pretreatment with BMS202. BMS202 led to greater p-Akt/Akt ratios. LIRI induced inactivation of Akt, which was further reinforced by inhibition of PD-1 (Fig. 5d, e). Similar results were obtained *in vitro*. Pretreatment with BMS202 partially reversed the OGD/R-induced upregulation of SHP1 and SHP2 (Fig. 5f–h), and it increased the p-Akt/Akt ratio (Fig. 5i, j).

### The SHP1/2-PI3K/Akt Axis Regulates M1-Type AM Polarization and Inflammation Induced by LIRI

**Fig. 6:**
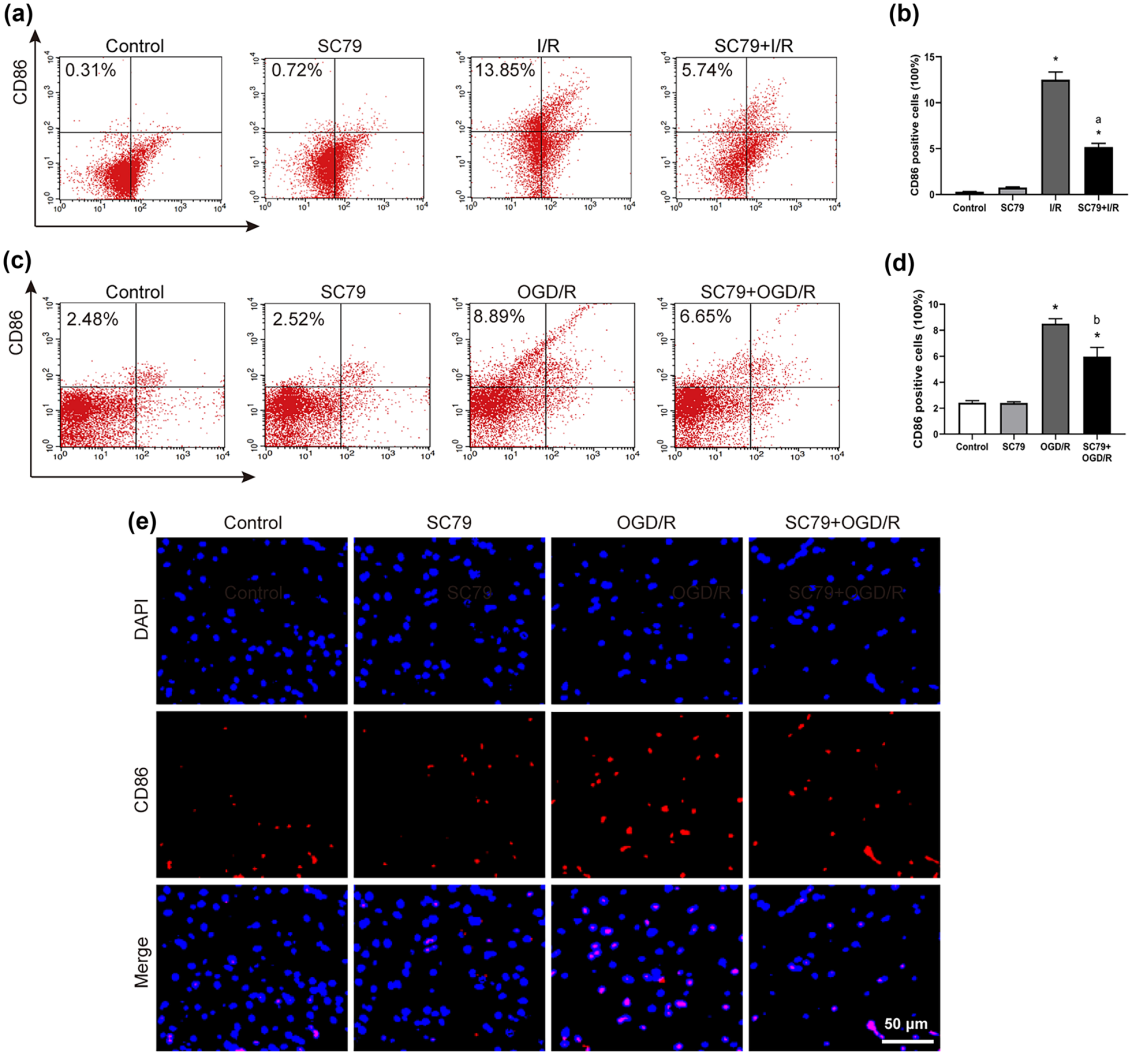

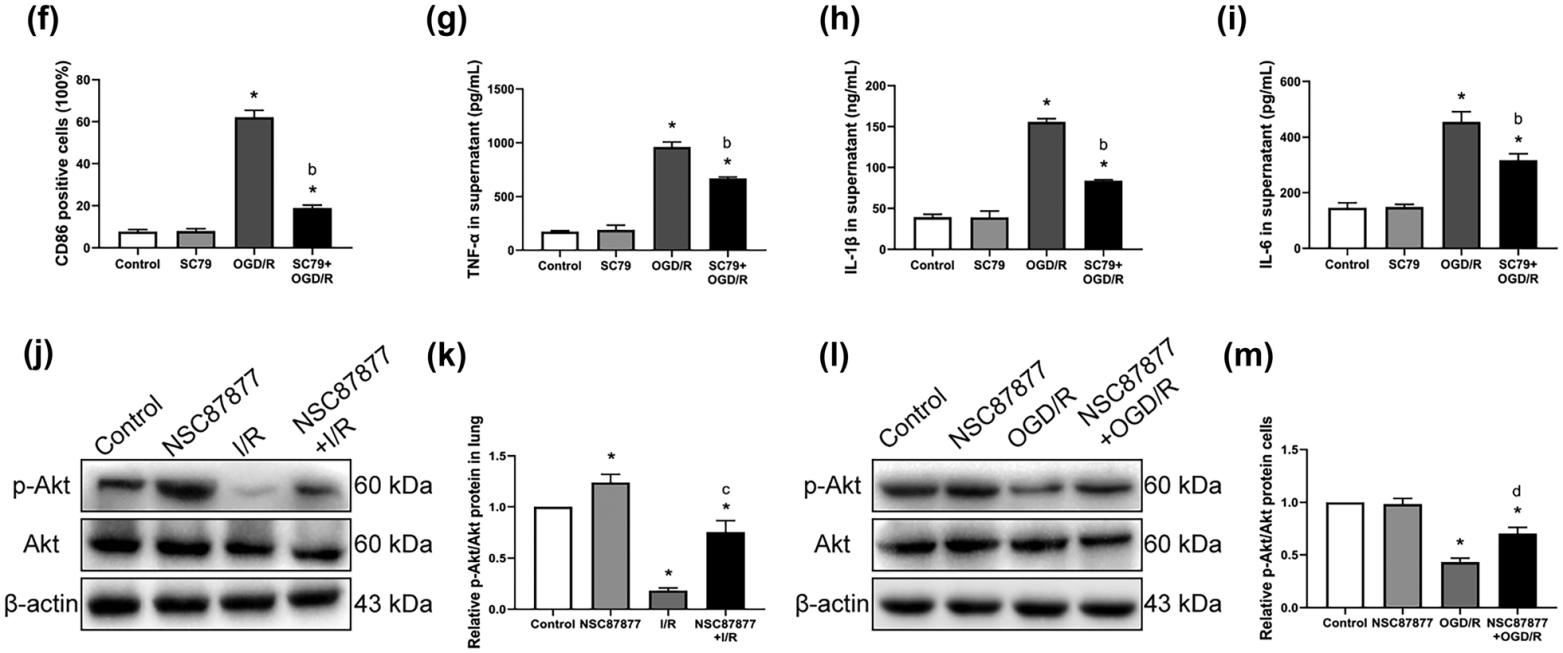
The SHP1/2-PI3K/Akt axis regulates M1-type AM polarization and inflammation induced by lung I/R. **a** and **b** Expression of CD86-positive AM cells in lung tissue by flow cytometry. **c** and **d** Expression of CD86-positive AM cells in NR8383 cells by flow cytometry. **e** and **f** Expression of CD86-positive AM cells in NR8383 cells by immunofluorescence. **g–i** Levels of TNF-α, IL-1β, and IL-6 in cell supernatant. **j** and **k** Western blot images and abundance of p-Akt and Akt in lung tissue. **l** and **m** Western blot images and abundance of p-Akt and Akt in NR8383 cells (*n* = 3 per group, mean ± SD, *P < 0.05 vs control group; ^a^*P* < 0.05 compared between I/R and SC79 + I/R group; ^b^*P* < 0.05 compared between OGD/R and SC79 + OGD/R group; ^c^*P* < 0.05 compared between I/R and NSC87877 + I/R group; ^d^*P* < 0.05 compared between OGD/R and NSC87877 + OGD/R group).

To determine the role of PI3K/Akt activity in macrophage polarization during LIRI, we tested the effects of the PI3K/AKTkt activator SC79 in rats subjected to I/R. Flow cytometry of lung samples revealed that I/R significantly increased the number of CD86-positive M1-type AMs, while SC79 exerted the opposite effect (Fig. 6a, b). Consistently, treating the OGD/R *in vitro* model with SC79 decreased the number of CD86-positive M1-type AMs (Fig. 6c–f). SC79 also significantly reduced levels of inflammatory factors induced by OGD/R (Fig. 6g–i).

Our results suggested an association between SHP1/2 and the PI3K/Akt pathway, which we verified by administering the SHP1/2 inhibitor NSC87877 to our LIRI rat and cell model. Pretreatment with this inhibitor significantly increased the p-Akt/Akt ratio, similar to the effects of PD-1 inhibition (Fig. 6j, k). NSC87877 also partially reversed the I/R-induced reduction in the p-Akt/Akt ratio (Fig. 6l, m).

### DISCUSSION

**Scheme 1:**
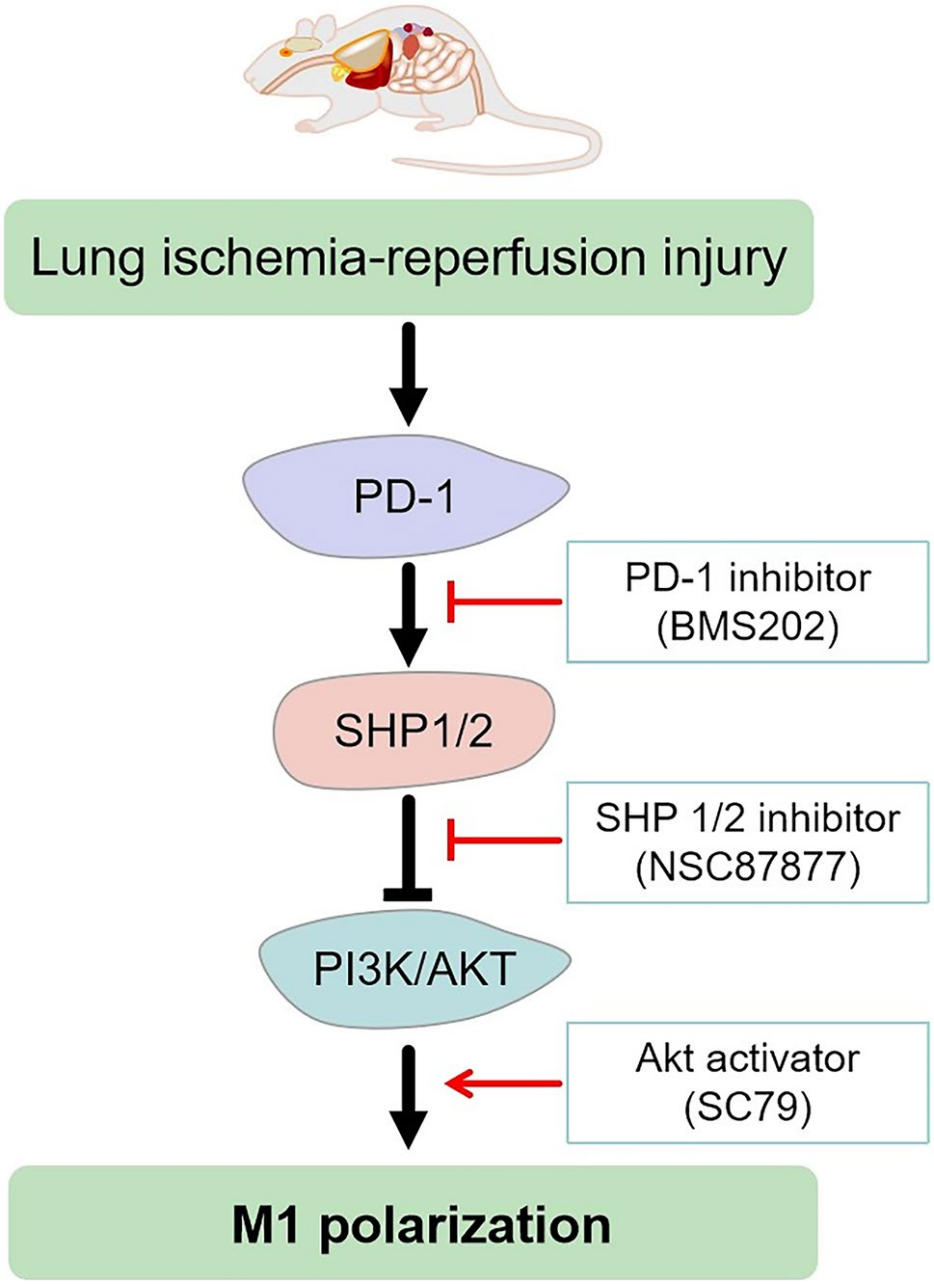
Schematic diagram of pulmonary ischemia–reperfusion in the rat model.

In this study, we explored the role of PD-1 in promoting M1-type AM polarization in the pathogenesis of LIRI. By inhibiting PD-1, we observed a significant reduction in expression of PD1 and polarization of M1-type AMs in our *in vivo* and *in vitro* models of LIRI. Furthermore, we found that PD1 may promote polarization of M1-type AMs by regulating the SHP1/2-PI3K/Akt axis (Scheme 1).

Macrophages play an essential role in the innate immune response. In the progression of LIRI, injury-associated molecular models activate AMs, a specific subset of lung macrophages [24]. AMs are capable of polarizing into different phenotypes and contribute substantially to lung inflammation. In the early stage of inflammation, AMs polarize to the pro-inflammatory M1 phenotype and secrete pro-inflammatory cytokines that damage tissue. However, when inflammation is in remission, AMs primarily polarize to the anti-inflammatory M2 phenotype and secrete anti-inflammatory mediators that promote tissue regeneration [8]. In this way, AMs maintain a dynamic balance between inflammatory and protective responses to injury. In animals with lipopolysaccharide (LPS)-induced acute lung injury, imbalance between the M1 and M2 phenotypes of AMs leads to inflammation following aftershock/resuscitation [30]. Therefore, regulating the polarization phenotype of AMs to reduce inflammation may be an effective way to treat LIRI.

One way to bias this polarization may be through targeting PD-1. However, PD-1 appears to play complex roles in I/R injury [11, 31, 32]. On one hand, activation of PD-1 signaling mitigates hepatic I/R injury by inhibiting T cell activation and Kupffer cell/macrophage function [33]; on the other hand, high PD-1 expression in cardiomyocytes after I/R results in inflammatory injury to myocardial tissues following reperfusion [13]. Downregulation of the PD-1/PD-L1 signal axis inhibits the apoptosis of AMs, reduces the secretion of inflammatory factors, and enhances lung protection in a mouse model of acute lung injury [34]. Our results support a more detrimental role for PD-1 expression after lung I/R, as inhibition of PD-1 with BMS202 significantly reduced inflammation and improved lung tissue and cell damage.

Consistent with other studies that the inhibitory effect of PD-1 was at least partly achieved by the recruitment of SHP1 and SHP2 [9, 35], we also found that inhibition of PD-1 reduced expression of SHP1 and SHP2, implying that the effects of PD-1 in LIRI involve recruitment of these two mediators. Binding of SHP1 and SHP to the ITSM domain of PD-1 inhibits activation of T cells [12]. In LPS-stimulated RAW264.7 macrophages, SHP2 binds PD-1 to inhibit downstream production of IL-12 [36]. Our findings indicate that in LIRI models, PD-1 recruits SHP1 and SHP2 to limit PI3K/Akt activity.

As a survival signaling pathway, the PI3K/Akt pathway is critical for macrophage activation, polarization, and apoptosis [16, 37]. Activation of the PI3K-Akt signaling pathway promotes M2 macrophage differentiation and may play an important role in inflammation resolution and tissue repair [38]. In addition, greater PI3K/Akt signaling protects the liver from I/R injury during liver transplantation by promoting M2 macrophage differentiation [39]. In contrast, inhibition of PD-1/PD-L1 increases the levels of Akt and mTOR and enhances the activity of AMs, thus improving the innate immune response to *Mycobacterium tuberculosis* in mice [40]. Although we did not measure numbers of M2-type AMs, we found that activating the PI3K/Akt pathway significantly reduced both M1-type AM polarization and lung I/R-mediated inflammatory cytokine production. These results suggest that stimulating PI3K/Akt signaling may be a way to reduce polarization of M1-type AMs and mitigate the inflammatory response in LIRI.

### CONCLUSION

Our results suggest that lung I/R significantly increases PD-1 expression and M1-type AM polarization. Inhibition of PD-1, inhibition of its binding partners SHP1 and SHP2, or activation of the PI3K/Akt pathway reduce polarization of M1-type AMs. Therefore, we propose that PD-1 promotes inflammation during LIRI through the recruitment of SHP1 and SHP2, which limits PI3K/Akt signaling. Future studies should examine whether deleting PD-1 from animal models of LIRI leads to greater PI3/Akt signaling and greater polarization to M2-type AMs. Our results suggest that inhibiting PD-1 may be a way to treat LIRI.

## Supplementary Information

Below is the link to the electronic supplementary material.

Supplementary file1 (ZIP 2172 KB)

## Data Availability

The data that support the findings of this study are available from the corresponding author upon reasonable request.
