# Supplementary figures and images for: Inhibition of PD-1 Alters the SHP1/2-PI3K/Akt Axis to Decrease M1 Polarization of Alveolar Macrophages in Lung Ischemia–Reperfusion Injury
Source: Inflammation. 2022 Nov 11;46(2):639–54. doi: 10.1007/s10753-022-01762-6 (PMC10024672; doi:10.1007/s10753-022-01762-6)

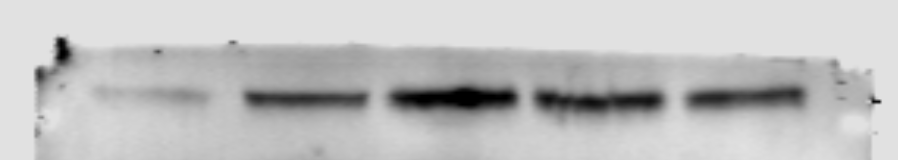

Supplement: Supplementary file 1 — Supplementary file1 (ZIP 2172 KB) [file 10753_2022_1762_MOESM1_ESM.zip › western blot/Figure 1/Figure 1F PD-1 blot.tif]

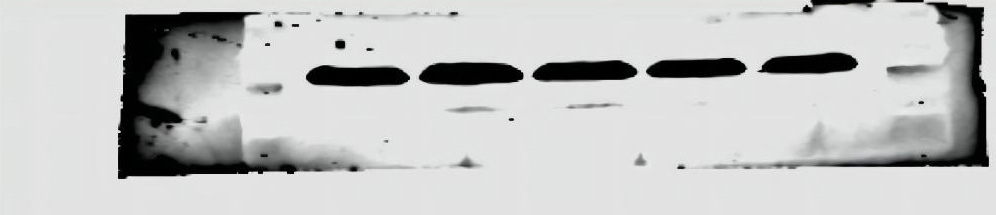

Supplement: Supplementary file 1 — Supplementary file1 (ZIP 2172 KB) [file 10753_2022_1762_MOESM1_ESM.zip › western blot/Figure 1/Figure 1F β-actin blot(1).jpg]

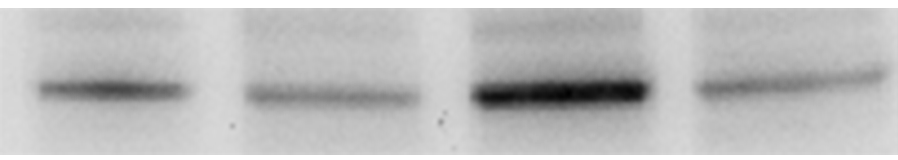

Supplement: Supplementary file 1 — Supplementary file1 (ZIP 2172 KB) [file 10753_2022_1762_MOESM1_ESM.zip › western blot/Figure 2/Figure 3H PD-1 blot 1.tif]

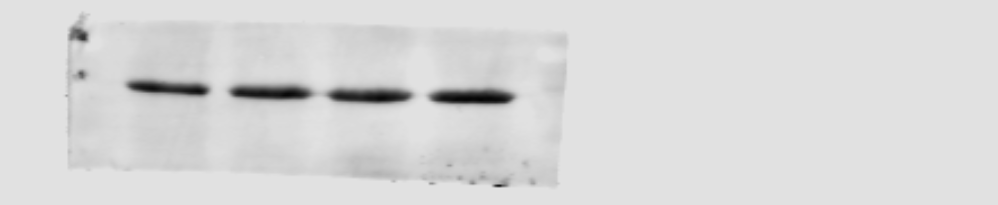

Supplement: Supplementary file 1 — Supplementary file1 (ZIP 2172 KB) [file 10753_2022_1762_MOESM1_ESM.zip › western blot/Figure 2/Figure 3H β-actin blot.tif]

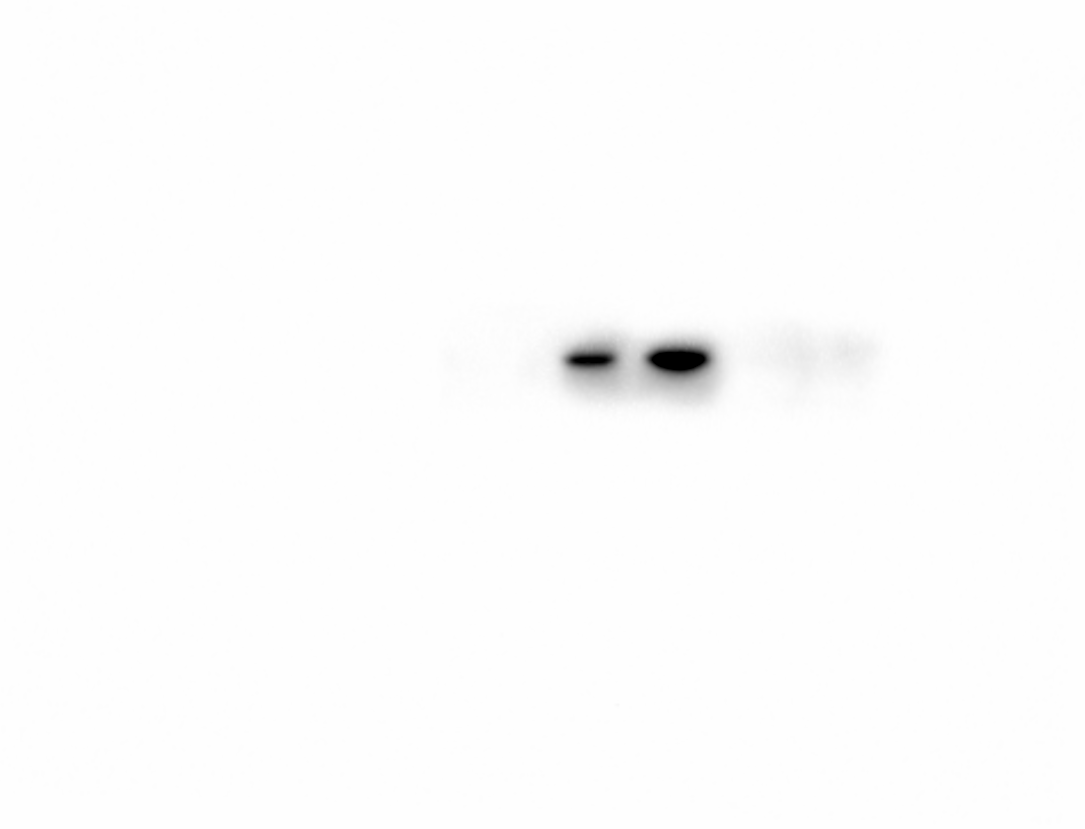

Supplement: Supplementary file 1 — Supplementary file1 (ZIP 2172 KB) [file 10753_2022_1762_MOESM1_ESM.zip › western blot/Figure 4/Figure 4a HIF-1α blot.tif]

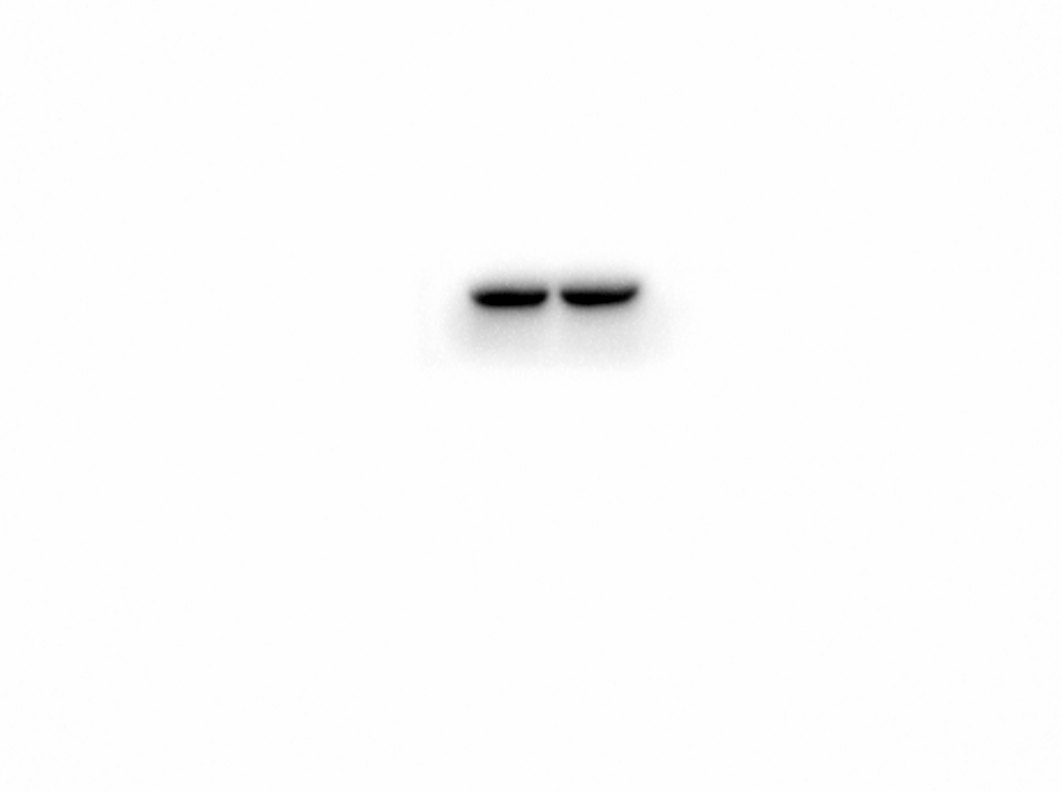

Supplement: Supplementary file 1 — Supplementary file1 (ZIP 2172 KB) [file 10753_2022_1762_MOESM1_ESM.zip › western blot/Figure 4/Figure 4a β-actin blot.tif]

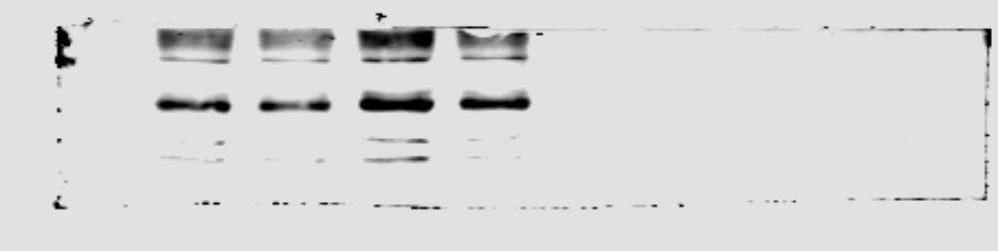

Supplement: Supplementary file 1 — Supplementary file1 (ZIP 2172 KB) [file 10753_2022_1762_MOESM1_ESM.zip › western blot/Figure 4/Figure 4c PD-1 blot.tif]

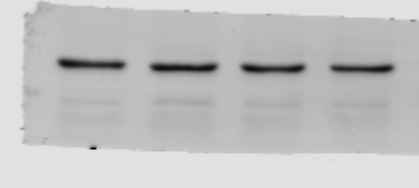

Supplement: Supplementary file 1 — Supplementary file1 (ZIP 2172 KB) [file 10753_2022_1762_MOESM1_ESM.zip › western blot/Figure 4/Figure 4c β-actin blot.tif]

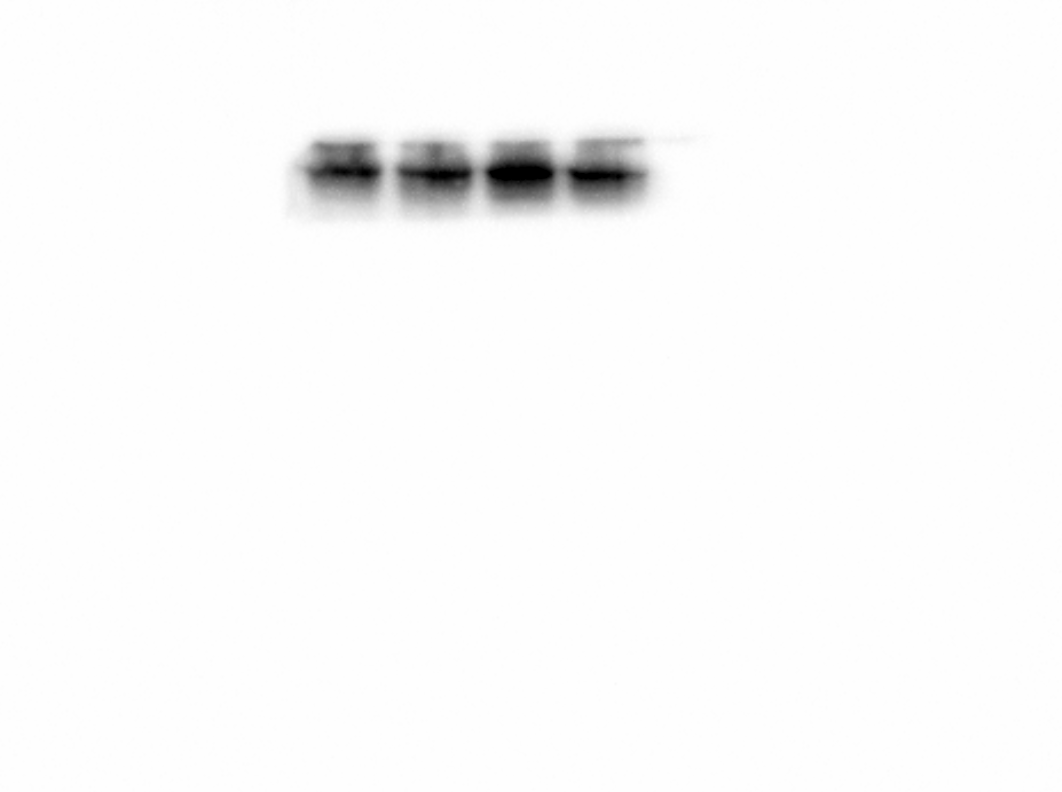

Supplement: Supplementary file 1 — Supplementary file1 (ZIP 2172 KB) [file 10753_2022_1762_MOESM1_ESM.zip › western blot/Figure 5/Figure 5a SHP1 blot.tif]

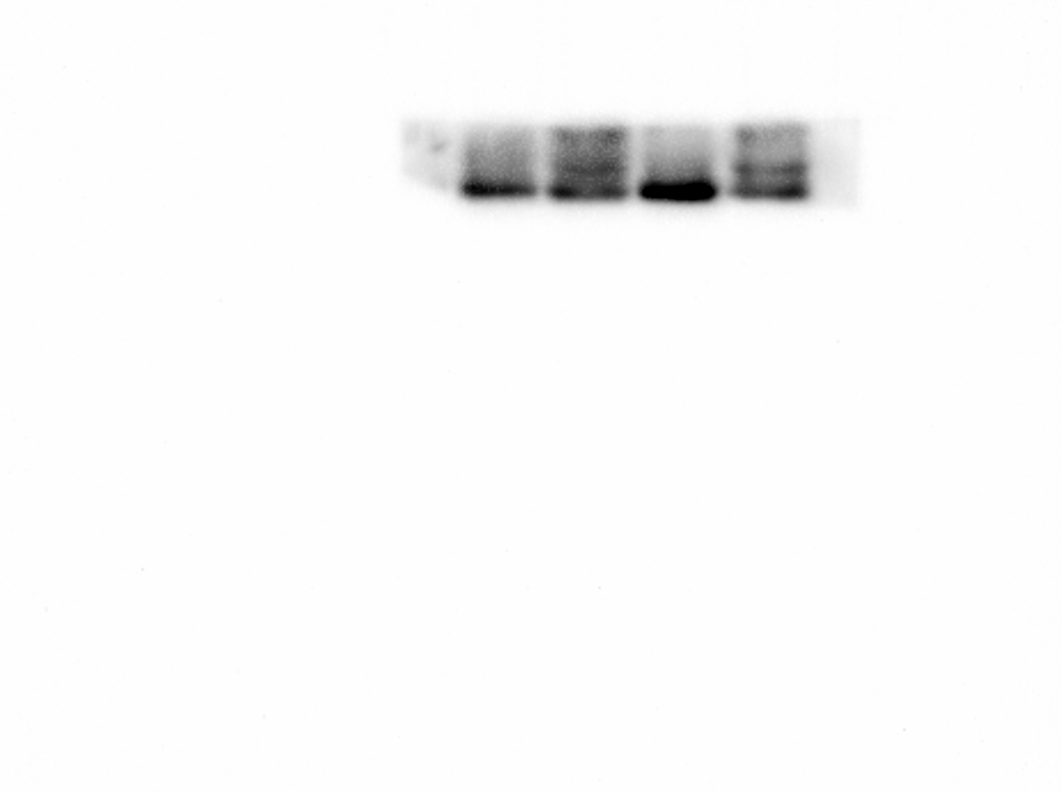

Supplement: Supplementary file 1 — Supplementary file1 (ZIP 2172 KB) [file 10753_2022_1762_MOESM1_ESM.zip › western blot/Figure 5/Figure 5a SHP2 blot.tif]

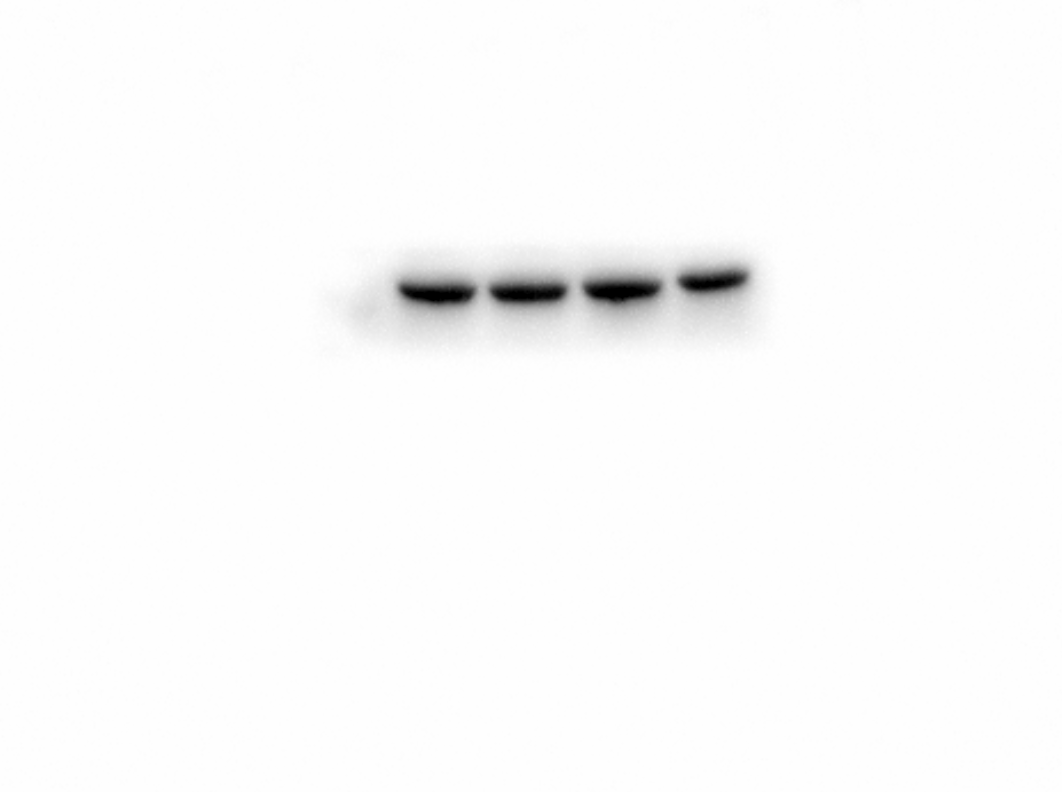

Supplement: Supplementary file 1 — Supplementary file1 (ZIP 2172 KB) [file 10753_2022_1762_MOESM1_ESM.zip › western blot/Figure 5/Figure 5a β-actin blot.tif]

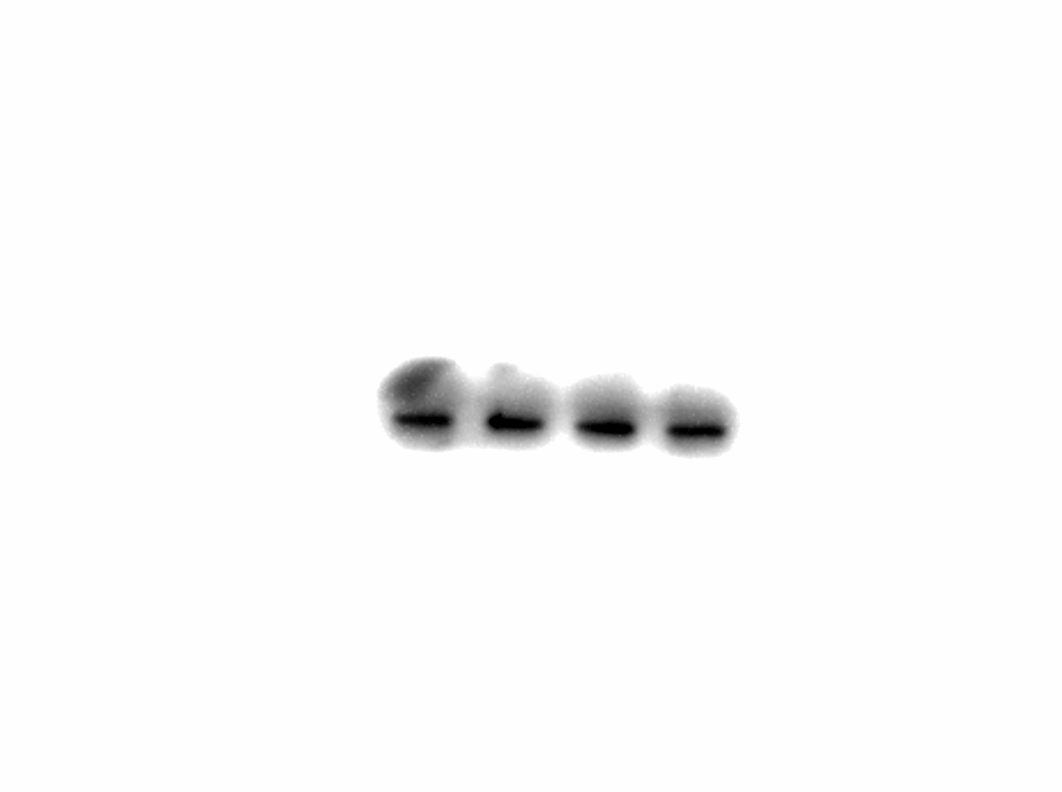

Supplement: Supplementary file 1 — Supplementary file1 (ZIP 2172 KB) [file 10753_2022_1762_MOESM1_ESM.zip › western blot/Figure 5/Figure 5d AKT blot.tif]

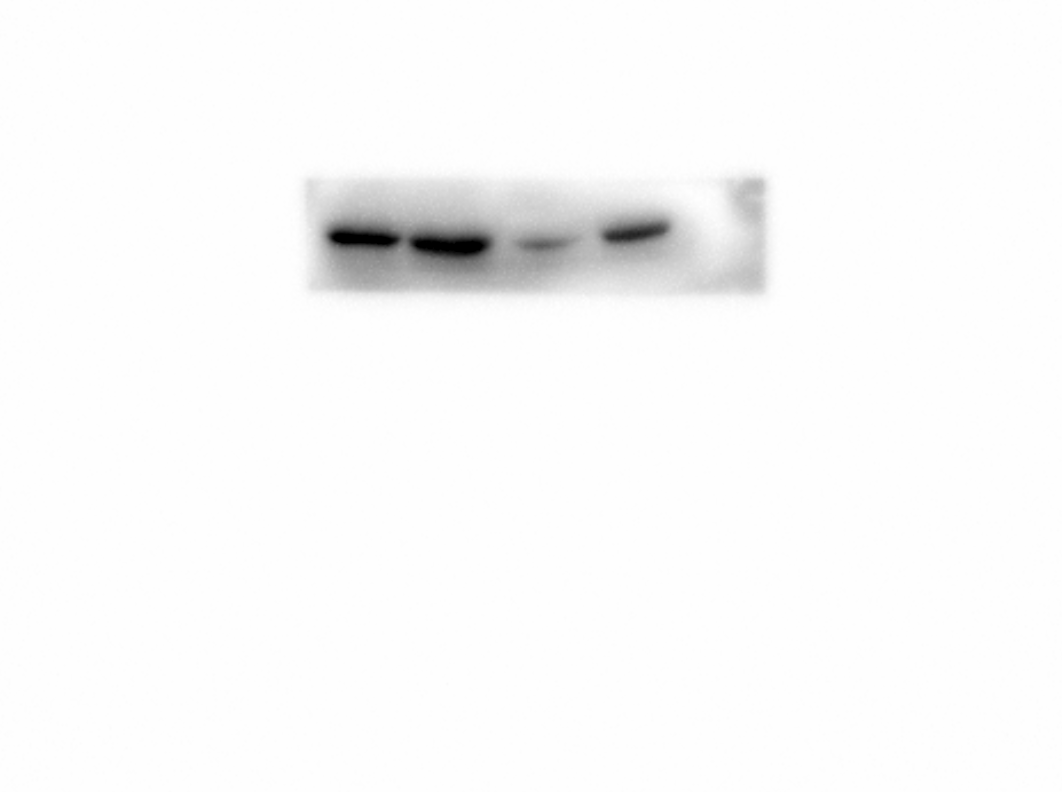

Supplement: Supplementary file 1 — Supplementary file1 (ZIP 2172 KB) [file 10753_2022_1762_MOESM1_ESM.zip › western blot/Figure 5/Figure 5d P-AKT blot.tif]

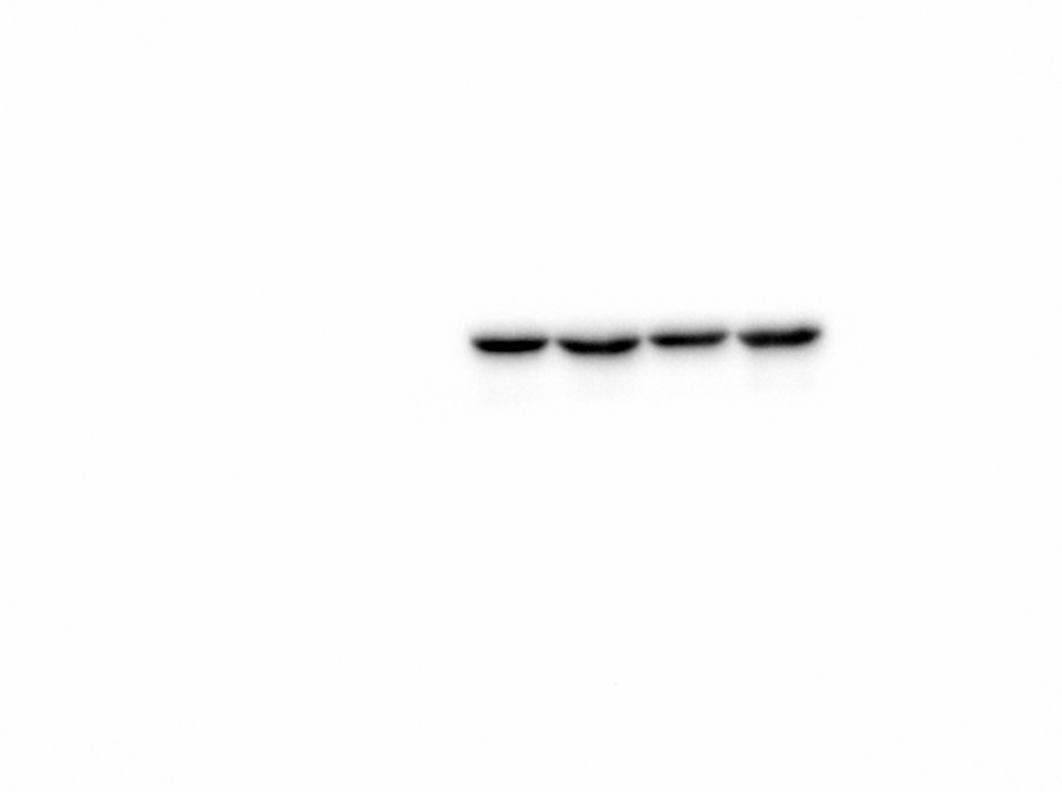

Supplement: Supplementary file 1 — Supplementary file1 (ZIP 2172 KB) [file 10753_2022_1762_MOESM1_ESM.zip › western blot/Figure 5/Figure 5d β-actin blot.tif]

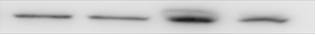

Supplement: Supplementary file 1 — Supplementary file1 (ZIP 2172 KB) [file 10753_2022_1762_MOESM1_ESM.zip › western blot/Figure 5/Figure 5f SHP1 blot.tif]

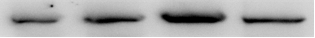

Supplement: Supplementary file 1 — Supplementary file1 (ZIP 2172 KB) [file 10753_2022_1762_MOESM1_ESM.zip › western blot/Figure 5/Figure 5f SHP2 blot.tif]

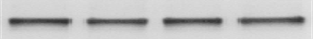

Supplement: Supplementary file 1 — Supplementary file1 (ZIP 2172 KB) [file 10753_2022_1762_MOESM1_ESM.zip › western blot/Figure 5/Figure 5f β-actin blot.tif]

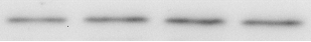

Supplement: Supplementary file 1 — Supplementary file1 (ZIP 2172 KB) [file 10753_2022_1762_MOESM1_ESM.zip › western blot/Figure 5/Figure 5i AKT blot.tif]

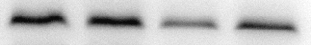

Supplement: Supplementary file 1 — Supplementary file1 (ZIP 2172 KB) [file 10753_2022_1762_MOESM1_ESM.zip › western blot/Figure 5/Figure 5i P-AKT blot.tif]

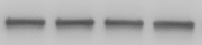

Supplement: Supplementary file 1 — Supplementary file1 (ZIP 2172 KB) [file 10753_2022_1762_MOESM1_ESM.zip › western blot/Figure 5/Figure 5i β-actin blot.tif]

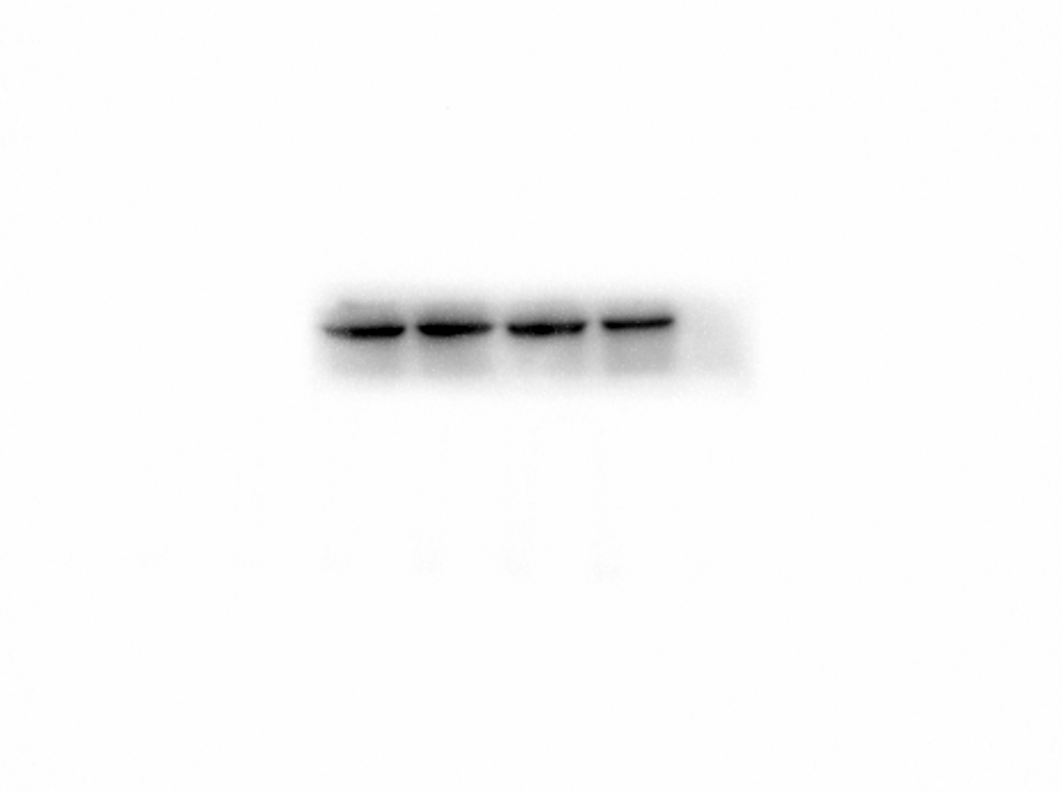

Supplement: Supplementary file 1 — Supplementary file1 (ZIP 2172 KB) [file 10753_2022_1762_MOESM1_ESM.zip › western blot/Figure 6/Figure 6l AKT blot.tif]

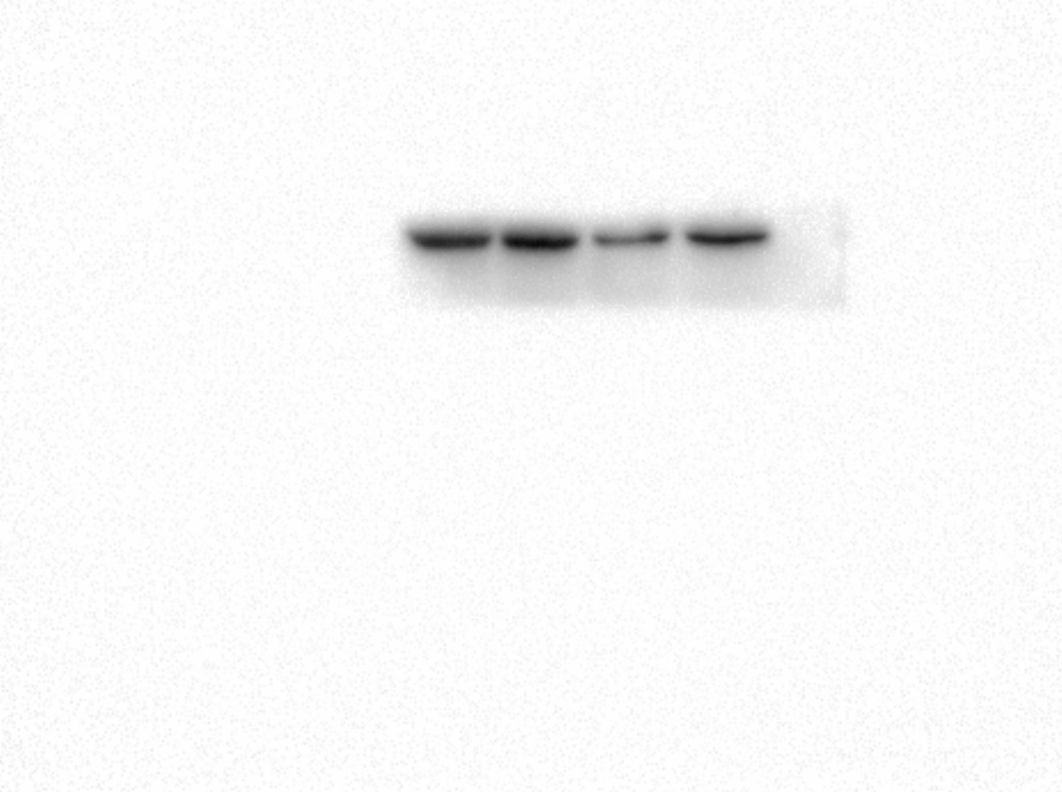

Supplement: Supplementary file 1 — Supplementary file1 (ZIP 2172 KB) [file 10753_2022_1762_MOESM1_ESM.zip › western blot/Figure 6/Figure 6l P-AKT blot.tif]

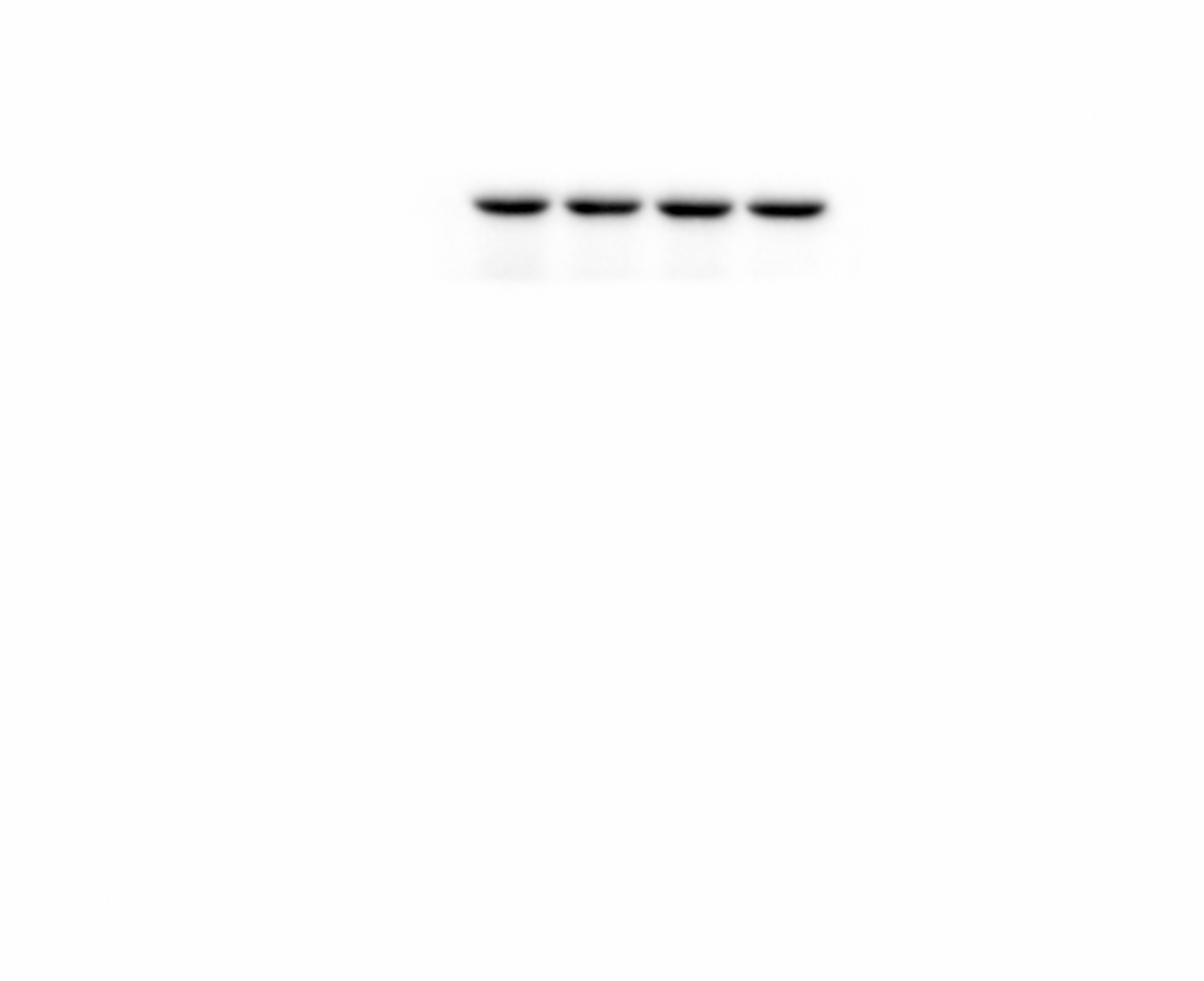

Supplement: Supplementary file 1 — Supplementary file1 (ZIP 2172 KB) [file 10753_2022_1762_MOESM1_ESM.zip › western blot/Figure 6/Figure 6l β-actin blot.tif]
